# Intervention of PKC-θ as an immunosuppressive regimen

**DOI:** 10.3389/fimmu.2012.00225

**Published:** 2012-08-02

**Authors:** Zuoming Sun

**Affiliations:** Division of Immunology, Beckman Research Institute of the City of Hope,Duarte, CA, USA

**Keywords:** PKC-θ, T cell activation, T cell differentiation, autoimmunity, allograft rejection

## Abstract

PKC-θ is selectively enriched in T cells and specifically translocates to immunological synapse where it mediates critical T cell receptor signals required for T cell activation, differentiation, and survival. T cells deficient in PKC-θ are defective in their ability to differentiate into inflammatory effector cells that mediate actual immune responses whereas, their differentiation into regulatory T cells (Treg) that inhibits the inflammatory T cells is enhanced. Therefore, the manipulation of PKC-θ activity can shift the ratio between inflammatory effector T cells and inhibitory Tregs, to control T cell-mediated immune responses that are responsible for autoimmunity and allograft rejection. Indeed, PKC-θ-deficient mice are resistant to the development of several Th2 and Th17-dependent autoimmune diseases and are defective in mounting alloimmune responses required for rejection of transplanted allografts and graft-versus-host disease. Selective inhibition of PKC-θ is therefore considered as a potential treatment for prevention of autoimmune diseases and allograft rejection.

## INTRODUCTION

T cells that are newly migrated out of thymus are naïve T cells incapable of mediating immune responses. Engagement of antigens with their T cell receptors (TCR) initiates the activation and differentiation programs that transform naïve T cells into inflammatory Th1, Th2, and Th17 effector cells or regulatory T cells (Treg), both of which participate in actual immune responses. The interference with molecules involved in T cell activation and differentiation is therefore considered an effective strategy to control the overwhelming immune responses that mediate autoimmunity and allograft rejection. However, most molecules involved in the processes of T cell activation and differentiation are not likely to be good targets for interference or inhibition because they also play essential roles in other cell types and signaling pathways. The ideal targets should meet at least the following criteria: (1) they selectively regulate T cell function; (2) small molecule inhibitors of them are easy to isolate or develop; (3) rather than inhibit, they actually promote Treg function. Ideally, inhibition of the target molecule should inhibit inflammatory T cells, and at the same time, enhance the suppressive function of Tregs. In this review, we summarize the evidence that supports PKC-θ as an excellent target for development of immunosuppressive agents.

## PKC-θ IS SELECTIVELY ENRICHED IN T CELLS

PKC-θ belongs to a family of serine/threonine kinases that consists of 12 different isoforms, each with distinct roles in the regulation of cellular functions (Newton, 1997). Members of this family can be divided into three subfamilies (Newton, 1997): conventional PKCs, including PKC-α, β, and γ, which are activated by Ca^2^^+^ and diacylglycerol (DAG); novel PKCs, including PKC-δ, θ, η, and ε, whose activation is dependent on DAG but is independent of Ca^2^^+^; and the atypical PKCs, PKC-ζ and λ, whose activation occurs independently of both Ca^2^^+^ and DAG. PKC-θ was first cloned as a novel PKC predominantly expressed in skeleton muscle (Osada et al., 1992; Chang et al., 1993) and found to be significantly enriched in hematopoietic compartments and skeleton muscle (Baier et al., 1994; Altman et al., 2000; Bauer et al., 2000). In hematopoietic compartments, PKC-θ is primarily expressed in T cells, but not B cells, and this has also been confirmed by *in situ* hybridization to mouse whole body sections (Bauer et al., 2000). The selective expression pattern of PKC-θ strongly suggests it plays a unique function in T cell compartments, and therefore may be a good immunosuppressive target for controlling T cell-mediated immunity. However, it is important to point out that T cells also express other isoforms of PKC (Bauer et al., 2000). Furthermore, although most studies have so far focused on PKC-θ function in T cells, there is evidence supporting that PKC-θ is also expressed and play a role in other tissues including muscle (Kim et al., 2004; Benoit et al., 2009; Paoletti et al., 2010), platelets (Nagy et al., 2009; Harper and Poole, 2010; Cohen et al., 2011), natural killer (NK) cells (Aguilo et al., 2009), and likely mast cells (Kempuraj et al., 2005). Therefore, inhibition or targeting of PKC-θ for immunotherapeutic treatments may also affect other tissues in addition to T cells.

## PKC-θ SELECTIVELY TRANSLOCATES TO IMMUNOLOGICAL SYNAPSE

PKC-θ attracted significant attention when it was shown among all the isoforms of PKC expressed in T cells, PKC-θ selectively translocates to the immunological synapse (IS), the stable cell–cell junction formed between T cells and antigen-presenting cells (Monks et al., 1997, 1998). The IS is a cluster of specialized membrane microdomains where TCR signaling molecules, including the TCR itself, are assembled (Grakoui et al., 1999). Formation of the IS is an active process that requires Lck-mediated signals to initiate re-organization of cytoskeleton (Morgan et al., 2001). Although there is still some controversy (Lee et al., 2002), it is generally believed that the IS serves as the platform that delivers integrated signals essential for T cell activation (Moran and Miceli, 1998). The IS consists of three major compartments: the central supramolecular activation cluster (cSMAC), the peripheral SMAC (pSMAC), and the distal SMAC (dSMAC; Barouch-Bentov et al., 2005; Dustin, 2009). The cSMAC was initially referred to as a signaling structure (Monks et al., 1998; Freiberg et al., 2002) and is located at the center of the IS. Around this center is the pSMAC, a ring of LFA-1/ICAM-1 co-localized with the cytoskeletal integrin linker talin (Monks et al., 1998). The outermost ring is the dSMAC, a zone enriched in the CD45 tyrosine phosphatase (Freiberg et al., 2002). Recently, it was demonstrated that the TCRs initially microcluster in the dSMAC, and then move through the pSMAC into the cSMAC, and is believed to be critical for the generation of continuous TCR signals that are required for T cell activation (Varma et al., 2006). PKC-θ is recruited to the junction between the cSMAC and pSMAC and co-localizes with TCRs in a CD28 co-stimulatory-dependent manner (Monks et al., 1997, 1998; Somersalo et al., 2004). Microscopic studies of IS have shown that T cells expressing PKC-θ periodically break open the pSMAC to create an asymmetric focal zone accumulation pattern that relocates to nearby areas where the pSMAC reformed (Sims et al., 2007). This periodic breaking of the symmetric pSMAC to form a polarized focal zone allows short bursts of migration, facilitating T cell interaction with multiple antigen presenting cells (Lindquist et al., 2004). This observation is also consistent with the asymmetric cell division theory that suggests that the IS leads to asymmetric cell division, a feature that is important for memory/effector differentiation of lymphocytes (Chang et al., 2007). A recent study has identified a unique region of PKC-θ, called the V3 domain, that is responsible for the selective translocation of PKC-θ to the IS (Kong et al., 2011). V3 was found to interact with the SH3 domain of Lck which is in turn, tethered to the phosphorylated tail of CD28 via its SH2 domain. The PKC-θ–Lck–CD28 interaction explains why PKC-θ recruitment to the IS depends on CD28 co-stimulation. However, in a different study the active kinase domain of PKC-θ was reported to be essential for PKC-θ translocation into the IS (Cartwright et al., 2011) and is not clear why there is a discrepancy. One possibility is that the two studies used different sources of T cells: primary T cells transduced with retrovirus and a D10 cell line. In contrast to conventional T cells, PKC-θ does not translocate to IS of Tregs. In fact it is actually sequestered away from the IS (Zanin-Zhorov et al., 2010), suggesting that the function of PKC-θ in Tregs is likely to be different from its functions in conventional T cells. Altogether, the fact that selective translocation of PKC-θ (but not other isoforms of PKC) to the IS is critical for T cell activation, strongly suggests it has unique functions in mediating TCR signals, and that selective inhibition of PKC-θ could specifically interfere with T cell function.

## PKC-θ MEDIATES TCR SIGNALS ESSENTIAL FOR T CELL ACTIVATION

TCR signals are initiated by activation of the Src family protein tyrosine kinase (PTK) Lck (Weiss and Littman, 1994), which leads first to recruitment of ZAP 70 and then the subsequent recruitment of the adaptor proteins LAT, SLP76, and VAV. LAT then recruits PLCγ1 (Clements, 2003; Berg et al., 2005) which catalyzes phosphatidylinositol 4,5-biphosphate into inositol triphosphate (IP3), a Ca^2^^+^ mobilizer and DAG, the PKC-θ activator (Weiss and Littman, 1994). In addition to DAG, PKC-θ activation seems to also require phosphorylation of threonine 538 (T538) in its activation loop (Liu et al., 2002; Lee et al., 2005). Previously, PDK1 was believed to phosphorylate T538 (Liu et al., 2002). However, a recent study indicates GLK is the upstream kinase responsible for T538 phosphorylation (Chuang et al., 2011). Upon activation, PKC-θ mediates the activation of NF-κB, AP-1, and nuclear factor of activated T cells (NFAT), critical transcription factors that are required for activation of the IL-2 gene (Sun et al., 2000; Pfeifhofer et al., 2003). Several adaptor proteins play a critical role in mediating PKC-θ-induced NF-κB activation including membrane-associated guanylate kinase (MAGUK), CARMA1, B-cell lymphoma 10 (Bcl 10), and mucosa-associated lymphoid tissue 1 (MALT1). Together with PKC-θ, these adaptors facilitate the activation of IKK, leading to the phosphorylation, ubiquitination, and degradation of IκB. Degradation of IκB releases NF-κB to the nucleus, where it participates in the activation of target genes essential for T cell activation (Weil et al., 2003; Lin and Wang, 2004; Weil and Israel, 2004). Studies using PKC-θ-deficient T cells or cells overexpressing the constitutively active PKC-θ or the catalytically inactive form of PKC-θ have demonstrated that PKC-θ also selectively activates the AP-1 signaling pathway in T cells (Sun et al., 2000). AP-1 is composed of c-Jun and c-Fos, which regulate many cellular events (Baier-Bitterlich et al., 1996). Although the exact mechanism responsible for PKC-θ-mediated activation of AP-1 is still unclear, several studies have provided some insight into this process. Ras and MAP kinases: JNK, ERK, and P38 are all involved in PKC-θ-mediated AP-1 activation (Baier-Bitterlich et al., 1996; Shaulian and Karin, 2002). Li et al. (2004) isolated a PKC-θ-interacting upstream MAP kinase, originally termed Ste20/SPS1-related proline and alanine-rich kinase (SPAK or PASK), and demonstrated that SPAK selectively interacts with PKC-θ and participates in PKC-θ-mediated activation of AP-1, but not NF-κB. Activation of T cells through the TCR also leads to IP3-mediated elevation of cytosolic [Ca^2+^]_i_ by inducing Ca^2^^+^ influx. Elevated intracellular Ca^2^^+^ ultimately leads to activation of the calcineurin phosphatase, which dephosphorylates NFAT, resulting in its translocation to the nucleus. Translocated NFAT cooperates with AP-1 to activate IL-2 expression and it has been shown that, in the absence of AP-1, NFAT activation can lead to T cell anergy (Macian et al., 2002). Several studies have shown that PKC-θ enhances the activation of NFAT by stimulating Ca^2^^+^ influx; TCR-induced Ca^2^^+^ influx, and NFAT activation is defective in T cells from *PKCθ*^− / −^ mice (Pfeifhofer et al., 2003; Manicassamy et al., 2006a). Although it is clear that PKC-θ regulates Ca^2^^+^ signals via stimulation of PLCγ1, it is not known how PKC-θ stimulates PLCγ1. The Tek kinase family member Itk may be the missing link. Itk-deficient T cells display defective Ca^2^^+^ influx and PLCγ1 activation (Liu et al., 1998), whereas overexpression of Itk leads to stimulation of PLCγ1 activity (Tomlinson et al., 2004). Therefore, it is possible that PKC-θ regulates PLCγ1 activation via Itk. Altogether, PKC-θ-mediated TCR signaling regulates multiple signaling pathways including NF-κB, AP-1, and NFAT that are all critical for T cell activation, which were clearly summarized in our previously published review articles (Manicassamy et al., 2006b; Kwon et al., 2010). Inhibition of PKC-θ is therefore expected to prevent T cell activation by blocking these pathways.

## PKC-θ ENHANCES T CELL SURVIVAL

Productive engagement of the TCR leads to T cell activation, resulting in cell proliferation and production of IL-2. Proliferating T cells especially during S phase of the cell cycle are susceptible to apoptosis (Boehme and Lenardo, 1993; Radvanyi et al., 1996). The TCR delivers signals that are required not only for stimulating proliferation but also for enhancing survival (Weiss and Littman, 1994; Boise et al., 1995). Such survival signals ensure the completion of the T cell activation process that is essential for differentiating naïve T cells into effectors that can mediate actual immune responses (Radvanyi et al., 1996). During T cell activation, survival of the T cells is enhanced by IL-2, which acts as an extrinsic survival factor. In addition, activated T cells substantially up-regulate Bcl-x_L_ that intrinsically increases resistance to apoptosis (Noel et al., 1996; Radvanyi et al., 1996; Van Parijs et al., 1996). We and others have shown that PKC-θ is required for the survival of activated T cells (Barouch-Bentov et al., 2005; Manicassamy et al., 2006a). *PKC-θ*^− / −^ T cells undergo apoptosis in response to TCR stimulation, which correlates with the reduced expression of NF-κB-dependent Bcl-x_L_. Forced expression of Bcl-x_L_ and Bcl-2 restores the survival of *PKC-θ*^− / −^ T cells and exogenous IL-2 can partially overcome the defective survival and proliferation of *PKC-θ*^− / −^ T cells. Similar to primary T cells, PKC-θ is also required for the survival of T cell lines such as Jurkat. It has been shown that PKC-θ promotes Jurkat survival by phosphorylating Bad and thereby inactivating its function (Villalba et al., 2001). However, primary T cells deficient in PKC-θ showed comparable levels of Bad phosphorylation to that of wild-type (WT) T cells (Barouch-Bentov et al., 2005), suggesting that the observed apoptosis of *PKC-θ*^− / −^ T cells is unlikely to be due to lack of Bad phosphorylation. Other pathways may also be involved in the apoptosis of *PKC-θ*^− / −^ T cells. For example, up-regulation of pro-apoptotic molecule Bim might be partially responsible for the observed apoptosis in *PKC-θ*^− / −^ T cells (Barouch-Bentov et al., 2005). Wan and DeGregori (2003) reported that inhibition of NF-κB leads to an increase in the expression of pro-apoptotic protein p73 . Since *PKC-θ*^− / −^ T cells are defective in NF-κB activation, it is possible that down-regulated p73 may contribute to the apoptosis of *PKC-θ*^− / −^ T cells. Because T cell survival ensures T cell-mediated immune responses, one of the mechanisms for PKC-θ to regulate immune responses is to enhance T cell survival and this is confirmed by our allograft rejection study discussed later in the review.

## PKC-θ PROMOTES THE DIFFERENTIATION OF NAΪVE T CELLS TO INFLAMMATORY Th17 CELLS

Differentiation of naïve T cells into specific T helper lineages is a critical checkpoint for controlling immune responses. Altered regulation of this checkpoint can lead to aggravated autoimmunity by overproduction of T helper cells that cause pathogenic inflammation. Similarly, allograft rejection also depends on the effectors that differentiate from allo-specific naïve T cells. *In vitro* stimulation of naïve CD4 T cells in the presence of appropriate cytokines up-regulates master transcription factors that instruct their differentiation into Th1, Th2, or Th17 inflammatory T helper cells (Dong, 2010). When stimulated with IL-12 or interferon-γ (INF-γ), naïve T cells express the T-box transcription factor T-bet as their lineage-specific transcription factor and differentiate into Th1 cells that secrete IFN-γ as their signature cytokine. In the presence of IL-4, naïve T cells express GATA3 as their lineage-specific transcription factor and differentiate into Th2 cells that secrete the signature cytokines IL-4, IL-5, and IL-13 (Murphy and Reiner, 2002). TCR stimulation in the presence of IL-6 and TGF-β leads to up-regulation of RORγt and differentiation of naïve T cells into Th17 cells that produce IL-17, IL-21, IL-22, and GM-CSF. We compared the differentiation of WT and *PKC-θ*^− / −^ T cell under Th1, Th2, and Th17 priming conditions *in vitro* to determine the function of PKC-θ in these processes and found that PKC-θ preferentially involved in the regulation of Th17 formation (Kwon et al., 2012). We showed that purified naive *PKC-θ*^− / −^ T cells were defective in Th17 differentiation, whereas Th1 and Th2 differentiation appeared normal. Activation of PKC-θ with PMA promoted Th17 differentiation in WT but not *PKC-θ*^− / −^ T cells. Furthermore, *PKC-θ*^− / −^ T cells had notably lower levels of Stat3, a transcription factor required for Th17 differentiation, and PMA markedly stimulated the expression of Stat3 in WT but not *PKC-θ*^− / −^ T cells. In contrast, activation of Stat4 and Stat6, which are critical for Th1 and Th2 differentiation, was normal in *PKC-θ*^− / −^ T cells. Forced expression of Stat3 significantly increased Th17 differentiation in *PKC-θ*^− / −^ T cells, indicating that reduced Stat3 levels were responsible for impaired Th17 differentiation and that Stat3 lies downstream of PKC-θ. Constitutively active PKC-θ or WT PKC-θ activated by either PMA or TCR cross-linking, stimulated the expression of a luciferase reporter gene driven by the Stat3 promoter. PKC-θ-mediated activation of the Stat3 promoter was inhibited by dominant negative AP-1 and IκB kinase-β, but stimulated by WT AP-1 and IκB kinase-β, suggesting that PKC-θ stimulates Stat3 transcription via the AP-1 and NF-κB pathways. Finally, conditions favoring Th17 differentiation induced the highest activation level of PKC-θ. Altogether, the data indicate that PKC-θ integrates the signals from the TCR activated with Th17 priming cytokines to up-regulate Stat3 via NF-κB and AP-1, which stimulate Th17 differentiation. The results are also consistent with the observation that PKC-θ-deficient mice are resistant to the induction of experimental autoimmune encephalomyelitis (EAE; Salek-Ardakani et al., 2005; Tan et al., 2006), which is a Th17-associated autoimmune disease. In contrast, and although *PKC-θ*^− / −^ mice have been reported to be defective in development of Th1 and Th2 immune responses, depending on the mouse model used (Marsland et al., 2004; Salek-Ardakani et al., 2004; Healy et al., 2006), our *in vitro* assays have shown that Th1 and Th2 differentiation is normal in the absence of PKC-θ. In addition to the apparently different priming conditions *in vitro* and *in vivo*, defects in other PKC-θ-regulated functions such as survival are likely to contribute to the overall defective Th1 and Th2 immune responses observed in *PKC-θ*^− / −^ mice *in vivo*. Although it is difficult to define which PKC-θ-regulated functions are responsible for the defective immune responses *in vivo*, we excluded the possibility that PKC-θ-regulated survival affected our differentiation assays *in vitro* by adding exogenous IL-2 which inhibits *PKC-θ*^− / −^ T cell apoptosis (Manicassamy et al., 2006a). Altogether, both *in vitro* and *in vivo* studies indicate that PKC-θ promotes the differentiation of Th17 cells that are associated with multiple autoimmune disorders (Huang et al., 2007).

## PKC-θ INHIBITS THE DIFFERENTIATION AND ENHANCES SUPPRESSIVE FUNCTION OF Tregs

Naïve CD4+ T cells can differentiate into either inflammatory effector T cells or Tregs (iTregs; Bettelli et al., 2006; Zhou et al., 2008), two distinct subsets of T cell helpers with opposite functions. A fine balance between these two opposing T cell types is required for a functional immune system. The activation of naïve T cells in the presence of TGFβ induces expression of Forkhead Box P3 (Foxp3), a master transcription factor instructing iTreg differentiation, and is also a marker for iTreg (Curotto de Lafaille and Lafaille, 2009). In contrast to iTregs, natural Tregs (nTregs) are not induced but develop in the thymus. The fact that naïve T cells can be differentiated into inhibitory iTregs suggests there is a therapeutic value for such a conversion in the treatment of autoimmunity. Our data has demonstrated that PKC-θ-mediated signals inhibit iTreg differentiation (Ma et al., 2012). We found that TGFβ-induced iTreg differentiation was enhanced in *PKC-θ*^− / −^ T cells or WT cells treated with a specific PKC-θ inhibitor, but was inhibited by the PKC-θ activator PMA or by CD28 cross-linking which enhances PKC-θ activation. Further, we showed that *PKC-θ*^− / −^ T cells had reduced activity of the AKT kinase, and that the expression of a constitutively active form of AKT in *PKC-θ*^− / −^ T cells restored their ability to inhibit iTreg differentiation. In addition, knockdown or over expression of the AKT downstream targets FoxO1 and FoxO3a was found to inhibit or promote iTreg differentiation in *PKC-θ*^− / −^ T cells respectively, indicating that the AKT-FoxO1/3A pathway is responsible for the inhibition of iTreg differentiation downstream of PKC-θ. Considering the positive role played by PKC-θ in the activation and differentiation of naïve T cells into inflammatory T effector cells (Altman et al., 2000; Sun et al., 2000; Pfeifhofer et al., 2003; Marsland et al., 2004), together the data indicate that PKC-θ is able to control T cell-mediated immune responses by shifting the balance between the differentiation of effector T cells and inhibitory Tregs.

In addition to Treg differentiation, a recent study also demonstrated a role for PKC-θ in the regulation of the effector function of Tregs (Zanin-Zhorov et al., 2010). Tregs inhibit inflammatory effector T cell function via cell contact-dependent and independent mechanisms (Sakaguchi et al., 2008). Such inhibitory functions of Tregs are important for establishing tolerance mechanisms required to prevent activation of autoreactive T cells that lead to autoimmunity (Sakaguchi et al., 2006; Shevach, 2006; Shevach et al., 2006; Miyara and Sakaguchi, 2007). The depletion of CD4+CD25+ Tregs from mice results in the development of widespread autoimmune/inflammatory diseases such as autoimmune gastritis, thyroiditis, type 1 diabetes, and inflammatory bowel disease. These autoimmune disorders are prevented by reconstituting the mice with CD4+CD25+ Treg cells (Sakaguchi et al., 1995; Singh et al., 2001), indicating a therapeutic potential for Tregs for the treatment of autoimmunity. To increase the efficacy of Treg-mediated inhibition, it is important to enhance the suppressive function of Treg. Inhibition of PKC-θ either by knockdown or a specific PKC-θ inhibitor has been shown to significantly boost the potential of Tregs to inhibit T cell activation (Zanin-Zhorov et al., 2010). However, in the presence of TGFβ neutralizing antibody, the PKC-θ inhibitor fails to enhance the suppressive function of Tregs, suggesting that inhibition of PKC-θ stimulates Tregs to produce the TGFβ that is responsible for inhibition of T cell activation. Interestingly, the suppressive function of Treg was also enhanced by inhibiting the activation of NF-κB, a critical downstream target of PKC-θ (Sun et al., 2000), indicating the possibility that PKC-θ inhibitor enhances Treg function by blocking activation of the NF-κB pathway. Furthermore, PKC-θ inhibitor-treated Tregs were more potent than untreated Tregs in preventing inflammatory colitis *in vivo* (Zanin-Zhorov et al., 2010), supporting the potential clinical application of PKC-θ inhibitors for Treg-mediated treatment of autoimmunity. Taken together, inhibition of PKC-θ can interfere with T cell-mediated immunity by inhibiting inflammatory T cell differentiation, by promoting Treg differentiation and by enhancing the suppressive function of Tregs.

## PKC-θ PLAYS A CRITICAL ROLE IN T CELL-DEPENDENT AUTOIMMUNITY

Due to the unique roles played by PKC-θ in the regulation of T cell activation and differentiation, PKC-θ is believed to be a potential drug target and pharmaceutical companies have developed highly specific PKC-θ inhibitors (Cywin et al., 2007; Mosyak et al., 2007). Mouse models of autoimmune diseases have been used to define PKC-θ function in T cell-dependent autoimmunity (Marsland and Kopf, 2008). Two independent studies have shown that *PKC-θ*^− / −^ mice were resistant to the induction of Th2-dependent lung inflammation in airway hyper responsiveness (AHR; Marsland et al., 2004; Salek-Ardakani et al., 2004), supporting a requirement for PKC-θ in Th2 type autoimmunity. In contrast, PKC-θ played a lesser role in the development of a similar lung inflammatory response mediated by Th1 cells (Salek-Ardakani et al., 2004), suggesting different functions of PKC-θ in Th1 and Th2 responses. However, PKC-θ was found to be essential for both methylated BSA and type II collagen-induced arthritis, a Th1-mediated autoimmunity disease (Healy et al., 2006). The results suggest that PKC-θ function is dependent on the model used. *PKC-θ*^− / −^ mice were reported by two different groups to be resistant to the development of Th17-mediated EAE, the mouse model for multiple sclerosis (Salek-Ardakani et al., 2005; Tan et al., 2006), indicating a requirement for PKC-θ in Th17-dependent autoimmunity.

The evaluation of PKC-θ function *in vivo* is complicated by several factors. First, PKC-θ function may be compensated for *in vivo*. Most *in vitro* assays clearly indicated essential role of PKC-θ in T cell functions (Manicassamy et al., 2006b). However, we found that the requirement for PKC-θ may be bypassed if T cells are stimulated by overwhelmingly strong TCR signals such as high concentrations of phorbol ester and ionomycin or anti-CD3/28 antibodies (unpublished data). It is therefore possible that PKC-θ function *in vivo* may also depend on the strength of TCR stimulation. In contrast to *in vitro* assays using purified T cells, *in vivo* immune responses involve many different types of cells that can produce factors to compensate for PKC-θ function. For example, one of the major signaling pathways that PKC-θ regulates is NF-κB. NF-κB can also be activated by PKC-θ-independent pathways such as TNFα and IL-1 (Sun et al., 2000). Many *in vivo* inflammation conditions produce TNFα and IL-1 inflammatory cytokines and these cytokines are likely compensate for PKC-θ function at least for the activation of NF-κB in T cells. Toll like receptors (TLR) can also activate the NF-κB pathway and it was indeed found that TLR-mediated signals can overcome the requirement for PKC-θ in T cell activation and the development of autoimmune myocarditis (Marsland et al., 2005). Second, autoimmune diseases usually involve more than one type of T helper cell. For example, both Th1 and Th17 responses are likely contribute to the development of EAE (Salek-Ardakani et al., 2005). In humans, it is even more difficult to specifically define the types of T helper cells involved in autoimmunity. Therefore, inhibition of PKC-θ may inhibit one type of T helper, but not other types of T helpers that can induce autoimmunity. Conversely, PKC-θ-mediated T cell differentiation is not the only PKC-θ function essential for the development of autoimmunity; PKC-θ also regulates other T cell functions including activation and survival. Therefore, the observed defects in the development of autoimmunity in *PKC-θ*^− / −^ mice are likely to be due to the disruption of several PKC-θ-regulated functions including those that have not yet been identified. However, despite the possible complications, the potential of PKC-θ as a drug target has been indicated by a trial for the treatment of psoriasis (Skvara et al., 2008). In this study, the clinical severity of psoriasis was reduced up to 69% after 2 weeks of treatment with a PKC inhibitor, AEB071 which inhibits multiple isoforms of PKC with strong specificity for PKC-θ, PKC-α, and PKC-β and lesser specificity for PKC-δ, PKC-ε, and PKC-η. Other clinical trials are expected because many companies have developed PKC-θ inhibitors. One of potential trials will be systemic lupus erythematosus, because patients with this disease show considerably enhanced activation of both PKC-θ and its potential upstream kinase GLK (Chuang et al., 2011).

## PKC-θ IS REQUIRED FOR NKT-MEDIATED AUTOIMMUNE HEPATITIS

Autoimmune hepatitis (AIH) results from the mistaken attack on healthy liver cells by an individual’s own immune system (McFarlane, 1999). In mice, acute hepatitis can be induced by treatment with concanavalin A (ConA), which causes rapid activation of CD1d-positive NK T cells. These activated NKT cells produce large amounts of cytokines that cause strong inflammation responsible for damaging liver tissues. Our research has shown that *PKC-θ*^− / −^ mice were resistant to ConA-induced hepatitis due to an essential requirement for PKC-θ during NKT cell development and activation. A dose of ConA (25 mg/kg) that was lethal to WT mice failed to cause death due to liver injury in *PKC-θ*^− / −^ mice (Fang et al., 2012). Correspondingly, the ConA-induced production of cytokines such as IFNγ, IL-6, and TNFα, which mediate the inflammation responsible for liver injury, were significantly lower in *PKC-θ*^− / −^ mice. In addition, upon stimulation with an NKT cell-specific lipid ligand, peripheral *PKC-θ*^− / −^ NKT cells produced lower levels of inflammatory cytokines than that of WT NKT cells, suggesting that activation of NKT cells requires PKC-θ. Our results suggest PKC-θ is an essential molecule required for activation of NKT cells to induce hepatitis (Fang et al., 2012), and thus, is a potential drug target for prevention of autoimmune hepatitis. NKT cells are also thought to be involved in liver injury induced by LPS, α-galactosylceramide (α-GalCer), *Salmonella* infection, chronic hepatitis C infection, and primary biliary cirrhosis (Ishigami et al., 1999; Kawano et al., 1999; Kaneko et al., 2000; Kato et al., 2000; Kim et al., 2002). Inhibition of PKC-θ is also likely to have therapeutic value in the treatment of liver injury in patients with these conditions.

## PKC-θ PLAYS A CRITICAL ROLE IN ALLOIMMUNE RESPONSES ESSENTIAL FOR TRANSPLANT REJECTION

Solid organ transplants that benefit end-stage organ failure patients are severely limited by the occurrence of rejection. Alloreactive T cells are critical targets for tolerance induction since they mediate the immune responses required for rejection. The alloreactive T cell pool is very large (Suchin et al., 2001), which explains why immune responses against allografts are at least two orders of magnitude stronger than immune responses against a specific antigen. Therefore, long-term tolerance to allografts is extremely difficult to establish. The initial evidence for requirement of PKC-θ in alloresponses came from the impaired *in vitro* mixed lymphocyte reaction of *PKC-θ*^− / −^ T cells (Sun et al., 2000). Injection of allogeneic cells into the footpad of PKC-θ-deficient mice provoked a significantly diminished local T cell response compared to WT mice similarly challenged, suggesting an essential role for PKC-θ in the allo-reaction *in vivo* (Anderson et al., 2006). We tested PKC-θ function in transplant rejection using a cardiac allograft model (Manicassamy et al., 2008). *Rag1*^− / −^ mice reconstituted with WT T cells readily rejected fully mismatched cardiac allografts, whereas, *Rag1*^− / −^ mice reconstituted with *PKC-θ*^− / −^ T cells failed to promote rejection, suggesting that PKC-θ is required for T cell-mediated allograft rejection. One of the important mechanisms responsible for establishing tolerance to allografts is to reduce the number of alloreactive T cells by inducing apoptosis (Wells et al., 1999). Since PKC-θ is required for survival of activated T cells (Manicassamy et al., 2006a), we therefore tested the role of PKC-θ-regulated survival in cardiac allograft rejection and demonstrated that the transgenic expression of Bcl-x_L_ in *PKC-θ*^− / −^ T cells was sufficient to restore cardiac allograft rejection (Manicassamy et al., 2008). This result suggests that apoptosis of alloreactive T cells in the absence of PKC-θ is responsible for the observed tolerance to cardiac allografts. Alloreactive T cells can be tolerized through anergy, suppression and deletion. Tolerizing mechanisms through anergy and Treg-mediated suppression are unlikely change the size of alloreactive T cell pool which is ready to destroy allografts. This is the problem for cyclosporin A (CsA), the most successful immunosuppressive drug used clinically so far. CsA prevents apoptosis of alloreactive T cells by inhibition of T cell activation (Li et al., 1999), resulting in accumulation of large amounts of alloreactive T cells that destroy allografts once the immunosuppressive drugs are discontinued (Li et al., 2001). Therefore, prevention of allograft rejection usually requires transplant recipients to take life-long immunosuppressive drugs, which can result in complications including infections and malignancy. Whereas, deletion induces tolerance by decreasing the number of alloreactive T cells via apoptosis, and thus avoids the potential risk of accumulating alloreactive T cells. In addition to an adoptive transfer model, we also tested cardiac rejection using intact *PKC-θ*^− / −^ mice. *PKC-θ*^− / −^ mice displayed delayed, but successful cardiac allograft rejection, suggesting there was some compensation for the missing PKC-θ function. Finally, a sub-therapeutic dose of anti-CD154 antibody or CTLA4-Ig, which was not sufficient to prevent cardiac allograft rejection in WT mice, prevented heart rejection in *PKC-θ*^− / −^ mice. *PKC-θ*^− / −^ mice treated with sub-therapeutic doses of Anti-CD154 or CTLA4-Ig also accepted donor-type second cardiac allografts but rejected third-party allografts (Wang et al., 2009). Thus, in combination with other treatments, the inhibition of PKC-θ allows long-term survival of cardiac allografts (Manicassamy et al., 2008; Wang et al., 2009).

In addition to heart rejection, the role of PKC-θ was also examined in a bone marrow transplantation (BMT) model (Valenzuela et al., 2009). BMT is used to replace damaged bone marrow with healthy stem cells or used as therapy for hematopoietic malignancies. In the latter case, allogeneic BMT boosts the patient’s immune system to aid in fighting against the cancer, which is called the graft-versus-leukemia (GVL) effect. However, graft-versus-host disease (GVHD), a potentially lethal consequence of BMT, limits the clinical application of this very effective treatment. Alloreactive donor T cells recognize the mismatched MHC of the recipient and undergo robust activation, expansion, and differentiation, resulting in GVHD, which causes severe damages to multiple tissues including gut, liver, skin, and kidney (Shlomchik, 2007). Immunosuppressive drugs are therefore needed clinically to prevent GVHD-induced damage. However, commonly used immunosuppressive drugs such as CsA and FK506 also inhibit the immune response against pathogens as well as tumors (GVL), and consequently limit the effects of GVL on the elimination of residual tumor cells (Reddy et al., 2005). The optimal immunosuppressive regimens are able to prevent GVHD, but also preserve the immune responses against infectious pathogens. Similar to WT mice, *PKC-θ*^− / −^ mice have the ability to respond to infection by the listeria bacteria and MCMV virus (Valenzuela et al., 2009). Moreover, *PKC-θ*^− / −^ mice survived the BMT procedure and did not develop GVHD, whereas majority of WT mice died from GVHD. More importantly, *PKC-θ*^− / −^ mice retained their ability to induce rejection of tumors. This study demonstrated that PKC-θ inhibitor-based immunosuppressive regimens are able to prevent GVHD but also preserve the protective immune response against infections and tumors.

## CONCLUSION

PKC-θ controls fundamental functions of T cells including activation, differentiation, and survival via NF-κB, AP-1, and NFAT pathways. PKC-θ also regulates T cell-mediated immune responses *in vivo* and selective PKC-θ inhibitors are believed to have the potential for clinical application in the treatment of autoimmunity and prevention of allograft rejection. However, PKC-θ function in T cell-mediated immune responses is dependent on the mouse models used. Therefore, the mechanisms involved in each of the diseases should be carefully examined. More questions need to be addressed prior to the clinical application of PKC-θ inhibitors including how the inhibition of PKC-θ affects the function of other tissues *in vivo*. It is encouraging to report that many pharmaceutical companies have developed selective PKC-θ inhibitors, and therefore many PKC-θ-regulated functions can be evaluated using these inhibitors instead of *PKCθ*^− / −^ mice, which have potential developmental caveat. With the availability of PKC-θ inhibitors, it is now possible to test their efficacy in mouse models of human autoimmune diseases including EAE and arthritis, which are likely to lead to clinical trials of PKC-θ-based treatments for human diseases.

## Conflict of Interest Statement

The author declares that the research was conducted in the absence of any commercial or financial relationships that could be construed as a potential conflict of interest.
